# The complete chloroplast genome sequence of *Gentianopsis grandis* (Harry Sm.) Ma (Gentianaceae) and phylogenetic analysis

**DOI:** 10.1080/23802359.2021.1947910

**Published:** 2021-07-09

**Authors:** Hailing Li, Jingjing Zhao, Nong Zhou

**Affiliations:** a College of Food and Biology Engineering, Chongqing Three Gorges University, Chongqing, China; b The Chongqing Engineering Laboratory for Green Cultivation and Deep Processing of the Three Gorges Reservoir Area’s Medicinal Herbs, Chongqing Three Gorges University, Chongqing, China

**Keywords:** *Gentianopsis grandis*, Gentianaceae, complete chloroplast genome, phylogenetic analysis

## Abstract

The high-throughput sequencing technology was used to sequence and assemble the chloroplast genome of *Gentianopsis grandis*, and we analyzed its structural characteristics and phylogenetic relationships. The complete chloroplast genome of *G. grandis* was 151,271 bp in length, consisting of a large single copy (LSC) region of 82,572 bp and a small single copy (SSC) region of 17,907 bp, which were separated by a pair of inverted repeat regions (IRs) of 25,396 bp. The annotation contained a total of 114 unique genes, including 78 protein-coding genes, 30 tRNA genes, four rRNA genes, and two pseudogenes. The phylogenetic analysis indicated the genus *Gentianopsis* was closely related to *Halenia* and *Swertia*.

*Gentianopsis grandis* (Harry Sm.) Ma is an annual or biennial herb that belongs to genus *Gentianopsis,* family Gentianaceae. *Gentianopsis* comprises 24 species in the world, and there are five species in China. *G. grandis* is distributed in the northwest of Yunnan province and the southwest of Sichuan province in China (Chen et al. 1999). Some species of *Gentianopsis* were extensively used for clinical treatment of conjunctivitis, hepatitis, nephritis, gastroenteritis, dyspepsia, fever and influenza in China (Lu et al. 2017). Nevertheless, most studies on *Gentianopsis* and its related genus have focused on describing chemical compositions, pharmacological activity and quantitative analysis using high performance liquid chromatography (HPLC) methods. DNA barcode was used to identify *Gentianopsis paludosa* from adulterant species (Xue and Li 2011). And only the chloroplast sequence of *G. paludosa* was submitted to Genbank. There are less researches about molecular biology of *Gentianopsis*. Here, we reported and characterized the complete chloroplast genome sequence of *G. grandis*, which can be used to reveal its phylogenetic relationships with other species of Gentianaceae.

Fresh and clean leaf materials of *G. grandis* were sampled from Songpan county, Sichuan province, China (30° 54’ 58.52’’ N, 103° 26’ 39.06’’ E), and the voucher specimen was deposited at the Herbarium of Medicinal Plants and Crude Drugs of the College of Pharmacy, Dali University (ZDQ17145). The total genomic DNA was extracted via the modified CTAB method (Doyle 1987; Yang et al. 2014). Genome sequencing was performed using Illumina Hiseq 2500 (Novogene, Tianjin, China) platform with the pair-end (2 × 300bp) library. About 3.61 Gb of raw reads with 10,222,390 paired-end reads were obtained from high-throughput sequencing. Raw data was filtered using Trimmomatic v.0.32 with default settings (Bolger et al. 2014). Subsequently, the trimmed reads were assembled into circular contigs using GetOrganelle (Jin et al. 2020) with *Swertia verticillifolia* (MF795137) as the reference. Finally, the complete chloroplast genome sequences of *G. grandis* were annotated in Geneious (version 11.0.2; https://www.geneious.com). The annotated chloroplast genome was submitted to the GenBank with an accession number MT591268.

The complete chloroplast genome of *G. grandis* is 151,271 bp in length with an overall GC content of 37.9%, exhibiting a typical quadripartite structure with a large single copy-region (LSC) of 82,572 bp, a small single copy-region (SSC) of 17,907 bp, and a pair of inverted repeats (IRa and IRb) of 25,396 bp. A total of 114 unique genes were annotated, including 78 protein-coding genes, 30 tRNA genes, four rRNA genes, and two pseudogenes (*rps 16* and *inf A*), of which 18 genes were duplicated in the inverted repeat regions. Among the annotated genes, 14 genes (*trnK-UUU*, *ndhA*, *trnl-GAU*, *trnA-UGC*, *petD*, *rpoC1*, *rpl16*, *atpF*, *petB*, *ndhB*, *trnG-UCC*, *rpl2*, *trnV-UAC* and *trnL-UAA*) contained one intron, whereas another two genes (*ycf3* and *clpP*) possessed two introns.

To investigate the phylogenetic position of *G*. *grandis*, 28 cp genomes were downloaded from the NCBI database and aligned using MAFFT version 7.149 (Katoh and Standley 2013). A neighbor-joining (NJ) tree was constructed using the MEGA v.7.0.26 (Kumar et al. 2016) with 1000 bootstrap replicates and three species (*Rauvolfia serpentine* MN746301*, Nymphoides coronate* MH201539 and *Menyanthes trifoliate* MH201540) were selected as an outgroup. The results revealed that the genus *Gentianopsis* was closely related to *Halenia* and *Swertia* (Figure 1). In summary, this study accurately revealed the phylogenetic position of *G. grandis* in the Gentianaceae, which would be beneficial to further phylogenetic studies on the related species or genera in the family.

**Figure 1.**
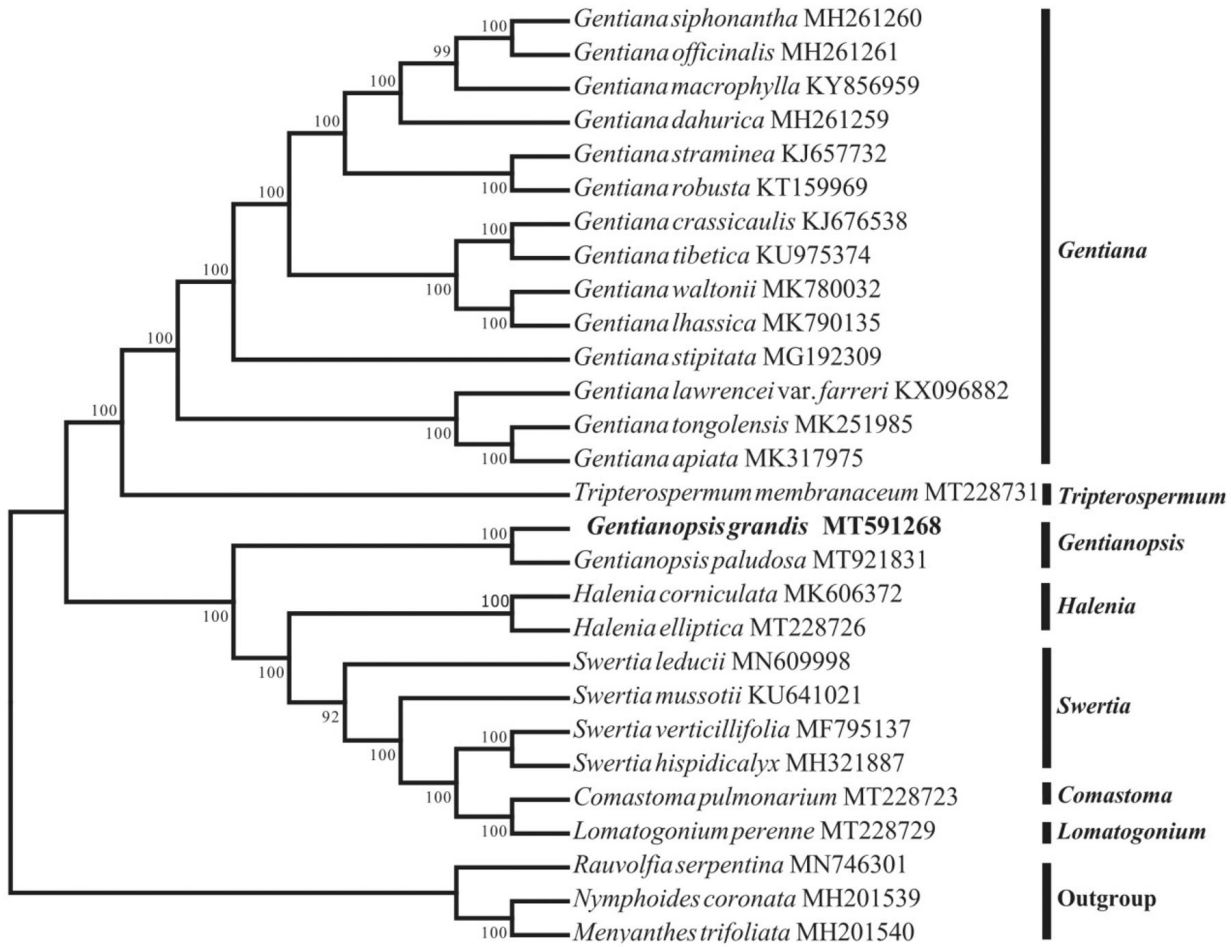
Neighbor-joining (NJ) phylogenetic tree of 25 species within the family Gentianaceae based on the complete chloroplast sequences using *Rauvolfia serpentina, Nymphoides coronate* and *Menyanthes trifoliate* as an outgroup.

## Data Availability

The genome sequence data that support the findings of this study are openly available in GenBank of NCBI at (https://www.ncbi.nlm.nih.gov/) under the accession no. MT591268. The associated BioProject, SRA, and BioSample numbers are PRJNA717403, SRR14076012, and SAMN18499221, respectively.
